# Structural ceramic batteries using an earth-abundant inorganic waterglass binder

**DOI:** 10.1038/s41467-021-26801-y

**Published:** 2021-11-11

**Authors:** Alan Ransil, Angela M. Belcher

**Affiliations:** 1 Department of Materials Science and Engineering, Massachusetts Institute of Technology, Cambridge, MA USA; 2 Koch Institute for Integrated Cancer Research, Massachusetts Institute of Technology, Cambridge, MA USA; 3 Department of Biological Engineering, Massachusetts Institute of Technology, Cambridge, MA USA

**Keywords:** Mechanical engineering, Batteries, Batteries

## Abstract

Sodium trisilicate waterglass is an earth-abundant inorganic adhesive which binds to diverse materials and exhibits extreme chemical and temperature stability. Here we demonstrate the use of this material as an electrode binder in a lay-up based manufacturing system to produce structural batteries. While conventional binders for structural batteries exhibit a trade-off between mechanical and electrochemical performance, the waterglass binder is rigid, adhesive, and facilitates ion transport. The bulk binder maintains a Young’s modulus of >50 GPa in the presence of electrolyte solvent while waterglass-based electrodes have high rate capability and stable discharge capacity over hundreds of electrochemical cycles. The temperature stability of the binder enables heat treatment of the full cell stack following lay-up shaping in order to produce a rigid, load-bearing part. The resulting structural batteries exhibit impressive multifunctional performance with a package free cell stack-level energy density of 93.9 Wh/kg greatly surpassing previously published structural battery materials, and a tensile modulus of 1.4 GPa.

## Abstract (web summary)

Structural batteries hold particular promise for decarbonizing the aviation industry. Here, the authors demonstrate that waterglass, an earth-abundant water-soluble silicate adhesive, can be used as a binder in structural batteries allowing them to both bear load and store electricity at the same time.

## Introduction

Structural energy storage aims to enable vehicle-level energy densities, exceeding those attainable using conventional designs by transferring mechanical load to multifunctional materials. This strategy offers improved vehicle range given constant active material chemistry, by reducing system mass as compared to designs based on single-function materials.^1–3^ Structural batteries hold particular promise for decarbonizing the aviation industry, where the low energy density of batteries compared with fossil fuels is a major barrier.^4,5^

Diverse approaches have been investigated in pursuit of practical structural energy storage systems. One strategy is to transfer the load to embedded commercial batteries^6,7^ as well as conventional battery electrodes and current collectors^8,9^. In developing multifunctional materials, structurally robust carbon and glass fiber manufacturing methods have been adapted to produce structural capacitor electrodes,^10–12^ electrolytes,^13–15^, and full devices.^16–21^ Such devices have achieved high performance as compared with other capacitor systems, but they typically exhibit energy densities under 10 Wh/kg. In developing multifunctional structural battery materials for improved energy density, both electrodes^22,23^ and separators^24–26^ have been fabricated and characterized. Integrating these components, full structural batteries have achieved energy densities up to 58 Wh/kg.^27–30^

Binder properties are key to the development of rigid multifunctional energy storage materials. In order to achieve high strength and stiffness during operation, the binder must adhere to electrode components and not soften when exposed to electrolytes. In addition, the binder must not block charge transport as this will decrease electrochemical performance. Organic polymer adhesives adapted from carbon fiber lay-up processes are frequently used^25,31–36^, which exhibit excellent mechanical properties but are impermeable to lithium ions. A multifunctional trade-off can be achieved with such binders, as the mechanical and electrochemical properties of the composite are tuned both chemically and by manipulating processing conditions^13,15,37^. Although future structural battery designs may incorporate solid electrolytes in order to further improve bulk material properties, such materials typically require thermal processing at temperatures incompatible with organic polymers^38^.

**Fig. 1:**
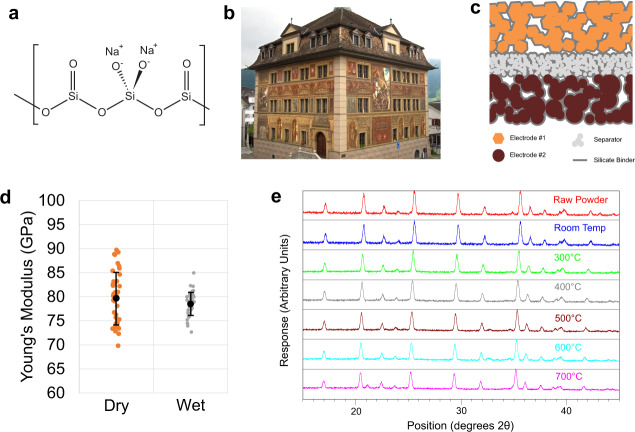
Use of silicate waterglass as a versatile adhesive. **a** The structure of sodium silicate waterglass. **b** Silicate-based mineral paint is chemically stable and able to withstand decades of exposure. **c** Structural ceramic battery (SCB) lay-up, using waterglass as a rigid binder within and between cell stack layers. **d** Silicate does not soften in the presence of EC:DMC electrolyte solvent (error bars: standard deviation). **e** In situ X-ray diffractogram demonstrates that silicate is stable when heated with Lithium Iron Phosphate up to 600 °C.

Here we report the use of waterglass as a robust binder for structural ceramic batteries (SCBs) overcoming the multifunctional trade-off between adhesion and ion transport. The primary constituent of waterglass is sodium trisilicate (Fig. 1a), a water-soluble earth-abundant inorganic compound that binds to a diverse range of materials with high-adhesion strength and exhibits extreme chemical and thermal stability^39,40^. These properties are leveraged across applications including use as a high-temperature refractory adhesive, a binder for exterior mineral paints able to withstand decades of weather exposure, (Fig. 1b), and as a cement sealant. In addition, silicate minerals are alkali conductors that have been employed as thin-film solid electrolytes^41^ and rate-enhancing active material coatings^42^. We show that this combination of adhesive and alkali transport properties renders waterglass well-suited as a multifunctional binder, able to function across all layers of a rigid battery stack (Fig. 1c). Furthermore, this chemistry enables a manufacturing process by which a flexible lay-up can be molded into the desired shape and then sintered to produce a load-bearing vehicle part.

## Results and discussion

### Structural binder characterization

Load-bearing structural battery electrodes must be rigid and remain so when exposed to electrolytes. Therefore, to assess the suitability of waterglass as a binder, nanoindentation was used to evaluate the effect of electrolyte wetting on the physical properties of dry and heat-treated films. The inorganic adhesive is two orders of magnitude stiffer than conventional polyvinylidene fluoride (PVDF) binder and does not soften when exposed to electrolyte solvent (Fig. 1d, *p* = 0.164), whereas PVDF becomes an additional order of magnitude softer.^43^ Furthermore, heat treatment improves mechanical performance at a temperature range compatible with typical active materials (Supplementary Fig. 1).

Silicate geopolymers undergo complex reactions with inorganic species facilitating binding. To assess interactions between this binder and active material, we performed X-ray diffraction of electrode films containing lithium–iron phosphate (LFP), waterglass, and conductive carbon heated in situ in an argon atmosphere. These samples showed no evidence of crystalline impurity phase formation up to 600 °C (Fig. 1e). A 21.1° impurity peak appearing at 700 °C corresponds to β-Na_2_Si_2_O_5_, a crystalline phase typically resulting from 400 °C heat treatment of amorphous waterglass deposits^44^. The increased crystallization temperature may be explained by alkali stabilization of the amorphous material^45,46^ through Li-Na cation exchange between the LFP active material and waterglass binder.

**Fig. 2:**
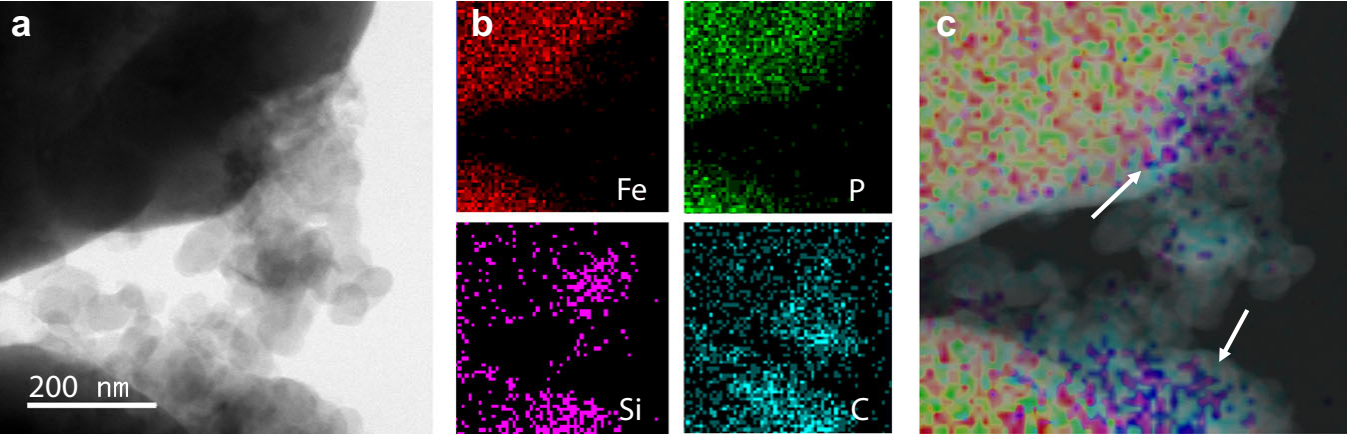
High-magnification elemental mapping of SCB electrode heated to 500 °C. **a** Bright-field TEM image of the interface between active LFP particle and conductive carbon. **b** Elemental mapping results confirm the location of silicon, carbon, and active materials. **c** Overlay demonstrating the localization of silicate at the active material interface.

High-resolution transmission electron microscopy of heat-treated electrode samples showed silicate binder at the interfaces between LFP and conductive carbon. Elemental mapping using energy-dispersive X-ray spectroscopy revealed silicon localized at this interface (Fig. 2, Supplementary Fig. 2), consistent with the waterglass-derived silicate serving as an inorganic binder. A comparison between electrode films dried at room temperature and those heated to 500 °C in an inert argon atmosphere showed no crystalline silicate phases in either condition, consistent with X-ray powder diffraction (XRD) data. In addition, the measured Na/Si elemental ratio decreased with heating (Supplementary Table 1), consistent with evidence for the exchange of alkali ions found during XRD tests.

**Fig. 3:**
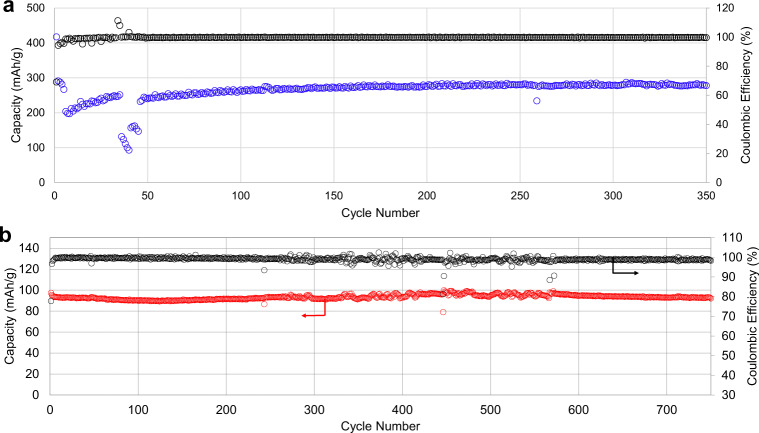
Long-term cycling of lithium half-cells with waterglass binder. **a** Graphite electrode showing a rate capability test followed by stable long-term cycling at C/5. **b** Lithium–iron phosphate electrode cycling test at 1C.

Lithium half cells made using waterglass-LFP electrodes demonstrated excellent cycling stability when formulated using negative (Fig. 3a) and positive (Fig. 3b) electrode materials. The cycling stability of waterglass-based graphite electrodes greatly exceeds that of previously reported samples employing sol-gel-derived silicate binders^47^. These tests demonstrate that geopolymers, chosen for use in other industries because their extreme stability, enable the production of electrochemically stable ceramic battery components.

**Fig. 4:**
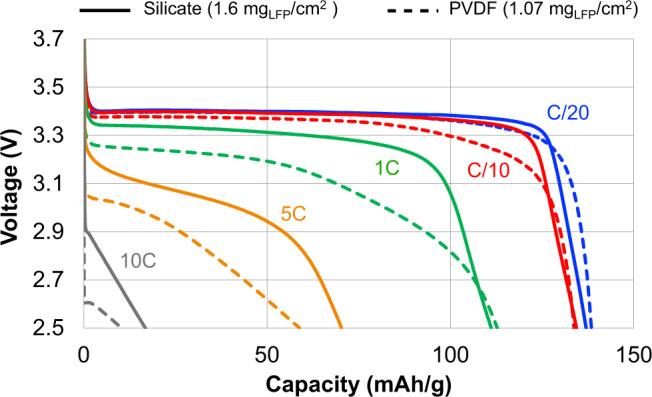
Rate comparison between LFP electrodes using silicate (1.6 mg_LFP_/cm^2^ loading) and PVDF (1.07 mg_LFP_/cm^2^ loading) binder in lithium half cells. Voltage curves of the sample produced using silicate demonstrate excellent charge transport.

Optimizing the heat treatment temperature of waterglass-based ceramic electrodes improved transport kinetics. Heat treatment of 290 °C, the minimum temperature sufficient to remove structural water,^44^ resulted in sloped half-cell voltage curves indicating slow lithium diffusion. Increasing the heat treatment temperature to 500 °C both restored the voltage plateau and improved the rate capability of these cells (Supplementary Fig. 3a). Improved lithium transport with heat treatment is consistent with Li-Na ion-exchange increasing the ionic conductivity of the waterglass-derived binder. In particular, as TEM did not reveal morphological changes with 500 °C heat treatment and XRD showed no new crystalline phases, this evidence suggests that changes in the chemistry of the amorphous silicate during heat treatment improve the transport kinetics of the electrodes. This is consistent with previous findings showing an improvement in ionic conductivity resulting from increased lithium content in silicate films used as a solid electrolyte in thin-film batteries^41^. Further supporting this conclusion, half cells made from slurries containing waterglass and graphite, in which no lithium was present during heat treatment, showed no effect of heat treatment temperature on rate capability (Supplementary Fig. 3b). In addition, while structural binders typically hinder ion transport^1,28^, we find that electrodes made using the ionically conductive inorganic binder exhibit no such trade-off. At loadings exceeding 1 mg_LFP_/cm^2^, the rate capability of electrodes made using a ceramic waterglass binder surpasses that of optimized electrodes made using conventional PVDF (Fig. 4). This comparison demonstrates that in combination, the adhesive and ionically conductive properties of heat-treated waterglass constitute a combination of properties well-suited to serve as the basis for high-performance SCBs. In particular, although replacing sodium with lithium in the binder may lead to future performance improvements, these tests show that common commodity waterglass adhesive forms a lithium-conductive binder in situ.

### Development of a shaped, load-bearing active stack

**Fig. 5:**
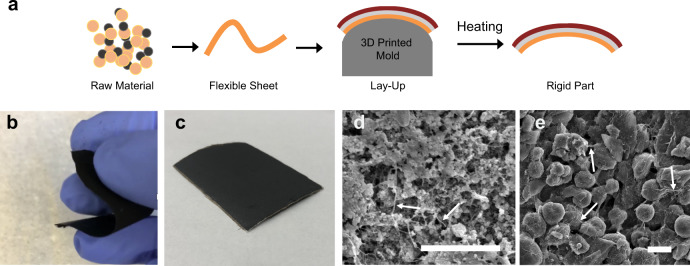
Layup based manufacturing process. **a** Flexible sheets are produced from active materials, formed into the desired shape, then sintered to produce the final part. **b** Flexible LFP sheet used in lay-up based fabrication of the full cell stack. **c** Full cell stack with curvature shaped using 3D-printed aluminum mold, scale bar 2 cm. **d**–**e** scanning electron micrographs of LFP (**d**) and graphite (**e**) electrodes with carbon nanofiber reinforcement, scale bars 2 µm.

We demonstrate that waterglass binder can be used as the basis for a lay-up manufacturing system producing rigid vehicle parts formed from flexible electrode and separator sheets (Fig. 5a). To produce sheets that can be molded into the desired shape, a temporary organic binder was added along with waterglass. This scalable process can be used to manufacture large, robust, free-standing flexible sheets that are easily handled (Fig. 5b, Supplementary Video 1) and formed into a full battery stack through a lay-up process. When the lay-up was heat treated in order to cure the waterglass adhesive, this process also removed the flexible binder. The result is a rigid multifunctional part of the desired shape. A 3d-printed aluminum mold was used to form electrode and separator sheets and maintain this shape during sintering. The mold could be used to precisely control sample curvature as is required for manufacturing applications in which shaped structural batteries will be integrated with a vehicle body (Fig. 5c). Such integration may use carbon fiber or glass fiber as a robust and scalable battery packaging material.

Sodium carboxymethyl cellulose (CMC) was used as the temporary organic binder, along with glycerol added as a plasticizer. Similar to waterglass, these additives are inexpensive, nontoxic, environmentally friendly commodity chemicals enabling electrodes to be produced from aqueous slurries. This polymer system furthermore provides the benefit that its stiffness can be readily tuned by varying the amount of glycerol plasticizer^48^ in order to accommodate electrode components and produce films with optimized mechanical properties to facilitate the lay-up process. In addition, CMC decomposes and glycerol boils at temperatures below the 500 °C used for waterglass curing,^49^ effectively removing the temporary binder system during the treatment process.

Several additives were used to further optimize multifunctional properties. Carbon nanofiber was added to positive and negative electrode sheets. Separator slurries were produced with silica particles and milled glass fiber as a filler, and coated onto thin fiberglass. The separator slurry composition was determined by using electrochemical impedance spectroscopy to assess the influence of structural binder content on lithium-ion diffusion (Supplementary Table 2, Supplementary Fig. 4, 5). The microstructures of rigid positive (Fig. 5d) and negative (Fig. 5e) electrodes resulting from heat treating these sheets show carbon nanofiber (arrows) and active materials. Lithium half-cell galvanostatic cycling tests of electrodes made using this composite system show capacity and cycling behavior comparable to those of the waterglass-based electrodes examined above (Supplementary Fig. 6). Following heat treatment, poly(ethylene oxide) was added to the full cell stack (Methods). This lay-up-based cell stack manufacturing procedure was performed entirely using aqueous slurries, contributing to the facile scalability and environmental friendliness of the process.

### Measuring structural battery performance

Determining a figure of merit for reporting the performance of structural battery materials is a complex endeavor^50,51^. Owing to the coupling between materials chemistry, manufacturing process, and vehicle design involved in developing load-bearing energy storage materials, a wide range of candidate figures of merit have been used. We surveyed 114 papers reporting advances in load-bearing structural energy storage materials and systems from 2008 to 2021 in order to assess the degree of consensus related to reported figures of merit (Supplementary Figures 7, 9–11, Supplementary Table 3, Supplementary Data 1). The majority (91%) of the papers focusing on structural electrolytes or separators reported an ionic conductivity, indicating a strong consensus on the importance of this metric. By contrast, manuscripts focusing on electrode materials for structural batteries showed no consensus as to the figure of merit used. The most common metric published among these papers was cycling data for custom-built full cells, which was reported by about a third (32%) of studies. Even this most common metric is not strictly comparable between papers because it follows no broadly accepted set of measurement or reporting standards. Custom cells vary widely in their construction and a detailed description of the mass of each cell component is frequently not available.

**Fig. 6:**
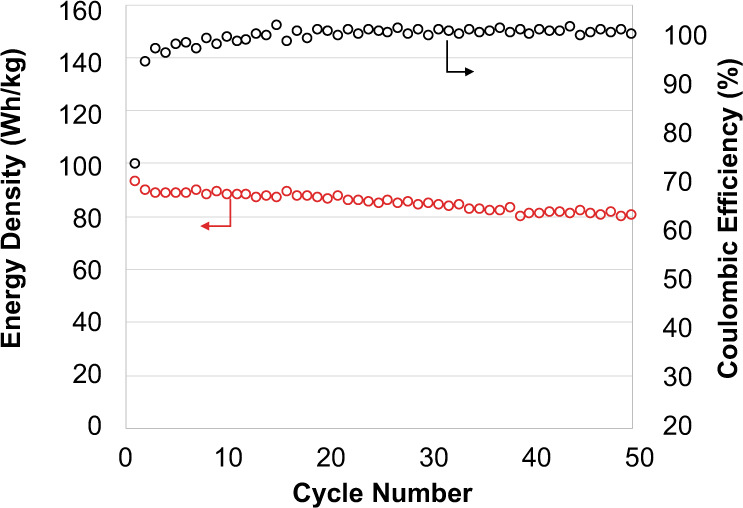
Structural ceramic battery (SCB) full cell cycling test at a C/5 rate. The initially low coulombic efficiency is due to prelithiation (methods). Energy density and coulombic efficiency indicate stable electrochemical performance over 50 cycles.

An optimal metric should separate the performance of full cells from cell packaging methods, which is a distinct manufacturing problem related to vehicle integration that few electrochemistry labs are equipped to study. We report the stack-level energy density of SCB coin cells (Fig. 6) along with a detailed breakdown of stack layer loadings (Supplementary Table 4). This data supply information necessary for a detailed projection of vehicle performance and for an assessment of the sensitivity of this projection to assumptions on vehicle manufacturing processes^2^.

This stack-level energy density metric makes explicit the emerging best standards in published structural battery literature. Although it is clear from recent papers that the performance of structural batteries is progressing rapidly, precise comparisons are difficult. For example, Moyer et al.^30^ recently published an energy density figure based on both active and structural device components. Asp et al.^29^ recently provided nearly but not directly comparable energy densities calculated based on both the device weight and based on active materials alone. With a detailed stack-level accounting of the mass of each device layer, direct comparisons would become possible.

**Fig. 7:**
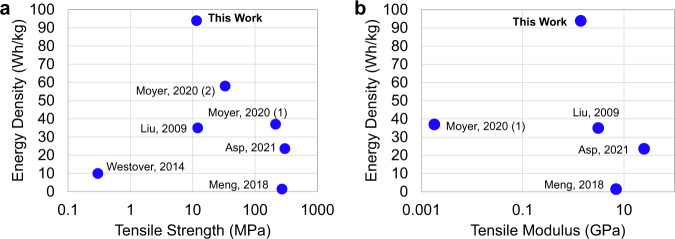
Multifunctional performance graph comparing the mechanical and energy storage performance of the SCB to work reported in the literature. Additional details are provided in Supplementary Table 3.

The full cell stack-level energy density of the SCB was reported based on the mass of each layer including electrodes, separator, and electrolyte, separating the electrochemical and structural performance of the SCB materials from packaging methods as described above. The masses used are given in Supplementary Table 4. The SCB energy density was 93.9 Wh/kg, (Fig. 6), which compares favorably to structural energy storage devices previously published (Fig. 7).

Similar to electrochemical metrics, the literature reveals very little consensus among researchers as to figures of merit for reporting the mechanical properties of structural batteries (Supplementary Figure 7). Because electrode and separator layers are all required for battery operation, the SCB cell stack is an irreducible unit of both energy storage and load-bearing structure. We, therefore, report cell stack tensile strength and stiffness, as these metrics are highly relevant for projecting structural battery performance in a vehicle. To conduct these tests, standard dogbone-shaped cell stack samples were fabricated according to the E8 ASTM standard for measuring the mechanical properties of electronically conductive materials and processed under the same conditions as samples for electrochemical measurements. We find that the SCBs exhibit a tensile strength of 11.5 MPa, with a tensile modulus of 1.4 GPa (Supplementary Figure 8).

A comparison of SCB electrochemical and structural properties to data from the literature is shown in Fig. 7. SCBs demonstrate remarkable multifunctional performance, showing a similar strength and stiffness compared with the median values demonstrated by other multifunctional energy storage composites and a greatly improved energy density.

### Future directions

This work leverages the unusual combination of properties possessed by silicate waterglass to produce rigid, load-bearing batteries of exceptional multifunctional performance. In addition, this novel binder material opens new avenues for the use of inorganic geopolymer adhesives in producing structural batteries. Geopolymers exhibit versatile chemistry, allowing the possibility of future optimizations in order to improve their adhesive and alkali transport properties. Because geopolymers are earth-abundant, water-soluble, and can be manufactured without toxic precursors, they are far more environmentally friendly than commonly used fluoropolymers. They are also produced cheaply at scale as commodity chemicals. Furthermore, these materials are compatible with extreme processing conditions such as the high temperatures used in producing inorganic solid electrolytes. Beyond allowing the practical lay-up manufacturing process demonstrated here, this high-temperature stability thus expands the space of processing conditions, electrode compositions, and manufacturing techniques available to explore new paradigms in structural energy storage.

## Methods

### Nanoindentation

Samples were made by casting a 2.65 wt% waterglass solution onto glass slides, allowing to dry for 2 h at room temperature, then heat treating in air at 90 °C for 2 h to remove residual moisture followed by a 2 h treatment at the stated temperature (all ramp times 30 min). Young’s modulus was measured using a Triboscan Nanoindenter with a diamond Berkovich tip. A 7 × 7 grid of 49 points was indented for each sample using displacement control mode with a peak displacement of 100 nm and a trapezoidal load function (10 s load, 3 s hold, 10 s unload). The initial, elastic section of the unloading curve was fit for Oliver Pharr analysis in order to obtain the reduced modulus *E*_*r*_. The sample modulus was calculated using the equation $1/{E}_{r}={\left(1-{{{{{{\rm{\nu }}}}}}}^{2}/E\right)}_{{{{{\rm{Tip}}}}}}+{\left(1-{{{{{{\rm{\nu }}}}}}}^{2}/E\right)}_{{{{{\rm{Sample}}}}}}$ using known values of the tip Poisson ratio *ν* = 0.07 and Young’s Modulus *E* = 1140 GPa, and using a Poisson’s ratio for the silicate of 0.18, from the literature^52^.

### X-ray diffraction

For in situ XRD measurements, electrode films were prepared using 85 wt% lithium–iron phosphate, 10 wt% Super P conductive carbon, and 5 wt% sodium silicate cast onto a glass slide. A Rigaku SmartLab instrument with a Cu K-α generator and a furnace attachment was used for the measurement. The generator was set to 45 kV and 200 mA. Samples were imaged using a parallel beam geometry with a soller slit of 2.5°, open PSA, a Ni fiter, and a D/teX Ultra linear position sensitive detector. Data were collected using a 2 theta/omega scan with a step size of 0.01, speed 7, and automatic attenuation. An argon flow of 200 cc/min was used to simulate electrode sintering conditions. 10 minute ramps were used between temperature set points.

### Transmission electron microscopy

For TEM tests, electrodes were made following the same procedure as for producing lithium half-cell electrodes (below), with the exception that samples were made with no heat treatment and with heat treatment. Heat treatment was in an Argon atmosphere, consisting of 2 hours at 90 °C followed by 2 hours at 500°C, with all ramps 30 min. Ethanol was added to the electrode surface and the sample was scraped with metal tweezers in order to suspend electrode particles in the liquid. 2 μl of suspension was placed on a carbon-coated Cu TEM grid (electron microscopy sciences). Imaging on an JEOL 2100 FEG microscope was done using a parallel illumination beam and 100 µm diameter condenser aperture. The microscope was operated at 200 kV and with a magnification in the range of 20,000 to 800,000 for assessing particle shape, size, and atomic arrangement. All images were recorded on a Gatan 2kx2k UltraScan CCD camera. STEM imaging was done using a high-angle annular dark-field detector with 0.5 nm probe size and 12 cm camera length. Energy-Dispersive Spectroscopy was measured in STEM mode using a X-Max 80 mm^2^ SSD detector and analyzed using Inca software.

### Rigid SCB electrodes

Electrodes were made based on a standard approach. A typical slurry was made by combining 33 wt% active material consisting of either lithium–iron phosphate (MTI Corp. EQ-Lib-LFPO-S21) or mesoporous carbon microbeads (MTI Corp. EQ-Lib-MCMB), 4 wt% Super P conductive carbon (TIMCAL), 14 wt% sodium silicate solution (Sigma Aldrich #338443), and 48 wt% deionized water. Super P was treated with 30% bleach solution by sonication for 30 mins, filtered, and dried in order to improve aqueous dispersion. Solids were ground in a mortar for 10 mins. Slurry was cast at 50 μm thickness directly onto a stainless steel coin cell spacer (Predd materials). Samples were dried in the air resulting in an electrode of ~15 μm thickness and then heat treated. Heat treatments consisted first of a ramp to 90 °C and a 2-hour hold at this temperature to fully dry the electrode. Subsequently, the electrodes were heated to a higher treatment temperature and held for 2 hours. All ramps were 30 minutes.

For comparison, lithium–iron phosphate electrodes were made according to a standard optimized recipe^53^. A solution was prepared by mixing 5.69 g NMP (Sigma Aldrich #M79603) with 0.241 g PVDF (MTI #EQ-Lib-PVDF) and heating to 80 °C to dissolve. Separately, 10 g LFP powder and 0.428 g Super P were ground with a mortar and pestle for 30 minutes. The slurry was cast onto aluminum foil and allowed to air dry, then pressed with a stainless steel roller to produce cathode sheets.

### Lithium half-cell tests

Coin cells were made using CR2023 casings prepared in an Argon glovebox maintained at [O2] and [H2O] below 0.1 ppm. Cell components were dried overnight at 90 °C in vacuum, and transferred to the glovebox without ambient exposure. The spacer with electrode adhered to it was placed on a spring in the coin cell casing. In all, 25 μl of 1 M LiPF6 in 1:1 v:v EC:DMC electrolyte (Sigma Aldrich #746711) and two pieces of Celgard 3501 separator were placed on top. A 9/16” diameter Li foil (Alfa Aesar #010769) was punched out and flattened with a stainless steel bar, and placed on top of the separator. The cell was crimped then removed from the glovebox. Rate tests were done using Biologic VSP and VMP3 battery testers. LFP electrodes were cycled between a voltage of 2.5 V and 4 V vs. Li, whereas graphite was cycled from 0 V to 2 V vs. Li. Three forming cycles were done at the C/20 rate based on the theoretical capacity of the active materials. The nominal capacity of the cells was taken to be the final lithiation (for positive electrodes) or delithiation (for negative electrodes) capacity of the cell. Subsequent tests were done based on this nominal rate. One rate test cycle typically consisted of C/10 charge (positive electrodes) or discharge (negative electrodes) followed by a test half cycle at a rate that varied. Three test cycles were done at each rate under investigation. All half-cell capacities were reported normalized by active material mass. Cycle life tests consisted of three forming cycles as described above, followed by charge and discharge at a symmetric rate using a Lanhe battery cycler. Rate was based on nominal capacity.

### Electrochemical impedance spectroscopy

EIS was performed with Biologic VMP3 and VSP instruments. CR2023 coin cells were made as constructed for half-cell tests, but using sequential layers of lithium foil, separator, sample, separator and lithium foil as shown in Supplementary Figure 5A as well as 50 μl of 1 M LiPF6 in 1:1 v:v EC:DMC electrolyte. Galvanic mode was used, applying a constant current of 0.4 mA and an oscillating signal of 100μA. The frequency was varied from 0.5 MHz to 1 Hz. In all, 10 points per decade were sampled.

### Flexible electrode and separator sheets

To make free-standing electrode films, a stock solution consisting of 3 wt% sodium CMC (Sigma Aldrich #419303), 6 wt% glycerol (Sigma Aldrich #G9012), 28 wt% sodium silicate solution (Sigma Aldrich #338443), and 87 wt% deionized water was prepared. To make a typical positive electrode film, 1.215 g lithium–iron phosphate (MTI Corp. EQ-Lib-LFPO-S21), 0.075 g Super P (TIMCAL), and 0.13 g carbon nanofiber (Pyrograf PR-19-XT-LHT) was ground in a mortar for ten minutes. Then 2.78 g of the stock solution was added along with 0.9 g of additional deionized water. The slurry was mixed for 10 minutes then cast at a thickness of 500 μm onto a Teflon sheet. An additional LFP formulation with no Super P, and otherwise identical composition, was fabricated in order to assess the effectiveness of carbon nanofiber as an electronically conductive additive (Supp. F). Negative electrodes were produced using a similar procedure, except that 0.65 g of mesoporous carbon microbeads (MTI Corp. EQ-Lib-MCMB), 0.1 g of carbon nanofiber, and 3.904 g of stock solution were used with no added water. Negative electrode films were cast onto glass sheets at a thickness of 150 μm. To make free-standing separator films, 1.941 g of stock solution was mixed for 10 mins with 0.547 g of 0.5 μm silica powder (Alfa Aesar #L1698514), 0.03 g of ¼” chopped glass fiber (Fibre Glast #30-A), 0.547 g of 1/16” milled glass fiber (Fibre Glast #C310329-A) and 0.1 g of deionized water. The slurry was coated onto high-density ultra-thin fiber-glass (110-μm thick) and allowed to dry. The sample was then flipped over and coated again, for a total of three coats.

### Full cell electrochemical tests

To make full cells, circular samples from flexible sheets were punched. Sizes were chosen in order to fit into a CR2023 coin cell. The separator and negative electrode samples were first wetted and pressed together in order to bond them. They were then heated in an argon atmosphere (2 hours at 90 °C, 2 hours at 500 °C, all ramps 30 min). The full cell stack was then assembled by wetting the positive electrode and adhering to the separator. This was pressed onto a coin cell spacer to flatten, and the entire stack was heated in an argon atmosphere (2 hours at 90 °C, 2 hours at 500 °C, all ramps 30 min). A 1 wt% poly(ethylene oxide) (Sigma Aldrich #372838, MW ~8,000,000) solution was added to the sample and allowed to dry. This was repeated, to achieve a total dry stack mass fraction of 2 wt% PEO. Silicone adhesive (Momentive RTV103) was applied around the edge of the separator and spacer to reduce the risk of breakage as the electrodes were subsequently handled.

Cell components were dried overnight at 90 °C in vacuum, and transferred to the glovebox without ambient exposure. In order to prelithiate the samples, coin cells were assembled with the full cell stack, a 9/16” diameter Li foil (Alfa Aesar #010769), and 30 μl 1 M LiPF6 in 1:1 v:v EC:DMC electrolyte (Sigma Aldrich #746711). A single piece of Celgard separator was placed in-between the lithium foil and negative electrode in order to enable the subsequent removal of lithium, and perforations in the separator allowed electrical contact between the lithium foil and the graphite. Prelithiathion and cycling tests were done on VSP and VMP3 battery testers. For prelithiation, cells were cycled five times at the C/20 rate based on the theoretical capacity of lithium–iron phosphate, at a voltage range from 2.5 V to 4.5 V. The final discharge was used to calculate the nominal capacity for cycling tests. Subsequently, the lithium foil was removed in an argon atmosphere and the cell was re-assembled. Full cell cycling was done from 2.5 V to 3.8 V at a C/5 rate based on the nominal capacity.

### Tensile tests

Dogbone-shaped structural battery layups were made following the ASTM E8 standard plan and tested using a Zwick BTC-EXMARCO.001 Mechanical Tester (Zwick/Roell). A 10 N load cell was used with an accuracy of 0.1 N. The machine was controlled using Test Xpert III software, based on the ASTM E8 standard. Grip-to-grip separation at test start was 6 cm, with a strain rate of 5 mm/min and a pre-load of 0.5 N.

## Supplementary information

Supplementary Information

Description of Additional Supplementary Files

Supplementary Data 1

Supplementary Movie 1

## Data Availability

The data sets generated during and/or analyzed during the current study are available in the “Structural Ceramic Batteries Using an Earth-Abundant Waterglass Binder” repository (Ransil, A. Structural Ceramic Batteries Using an Earth-Abundant Waterglass Binder. (2021). Available at: osf.io/9ntsf.)
