# Supplementary material for: Structural ceramic batteries using an earth-abundant inorganic waterglass binder
Source: Nat Commun. 2021 Nov 11;12:6494. doi: 10.1038/s41467-021-26801-y (PMC8585950; doi:10.1038/s41467-021-26801-y)
Supplement: Supplementary file 1 — Supplementary Information [file 41467_2021_26801_MOESM1_ESM.pdf]

Supplementary Information

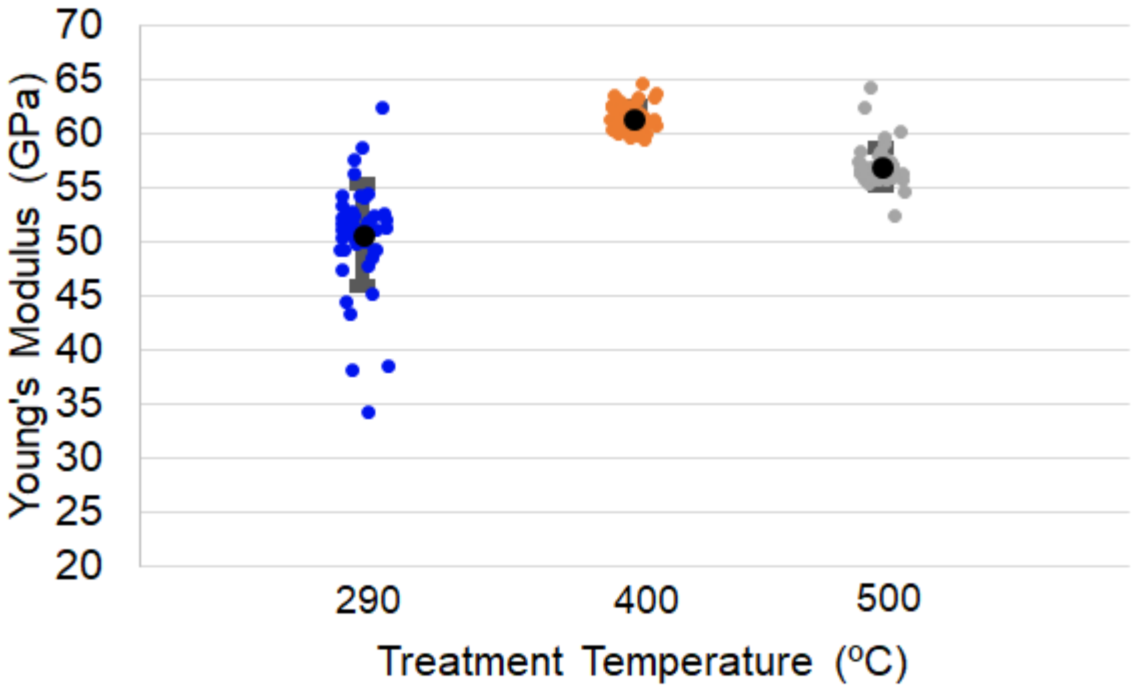

Supplementary Figure 1: comparison of Young's Modulus of silicate films heat treated at different temperatures (error bars: standard deviation).

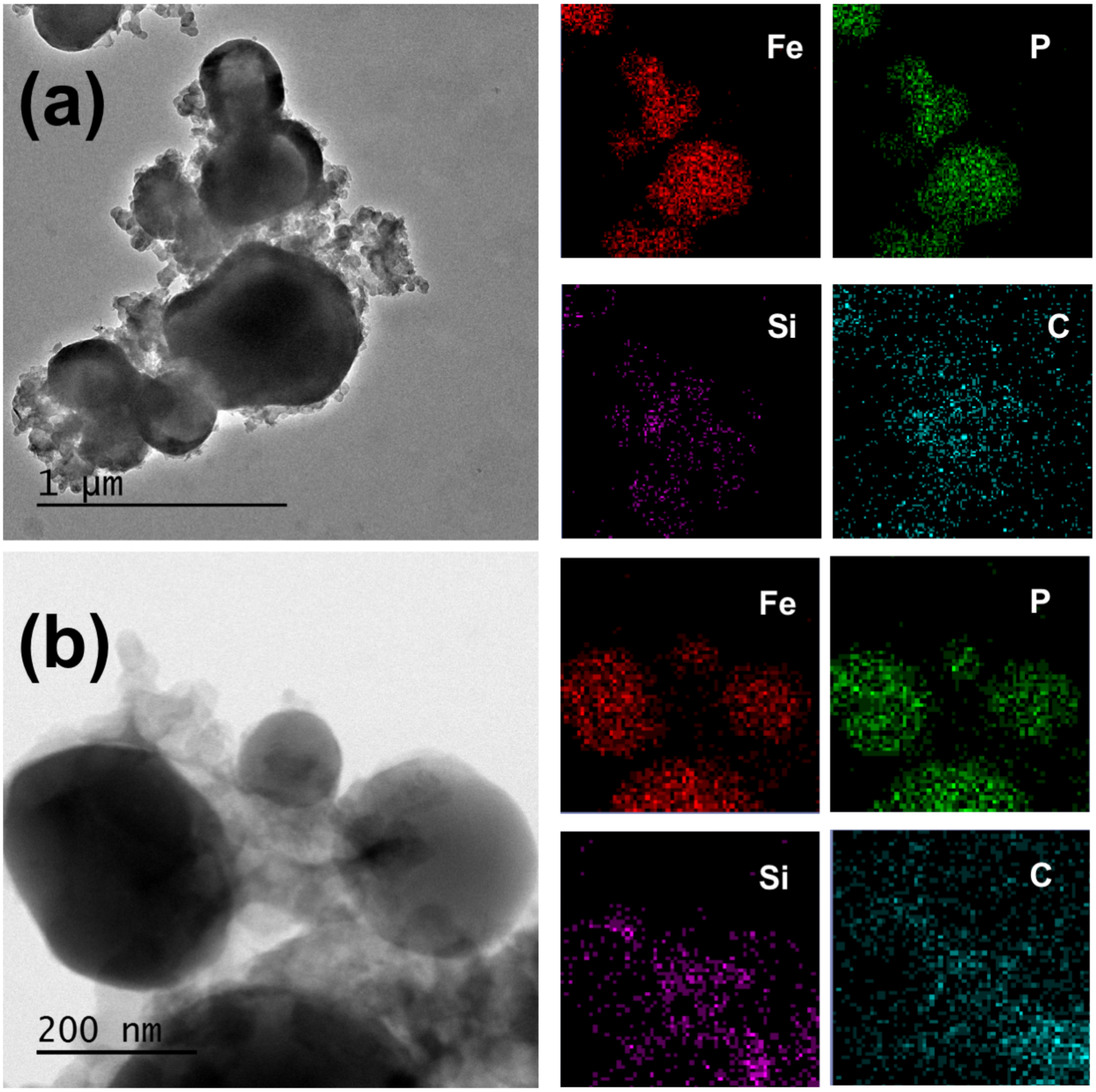

Supplementary Figure 2: TEM images and elemental mapping of electrode samples with no heat treatment (A) and heat treatment to 500ºC (B) as described in the methods.

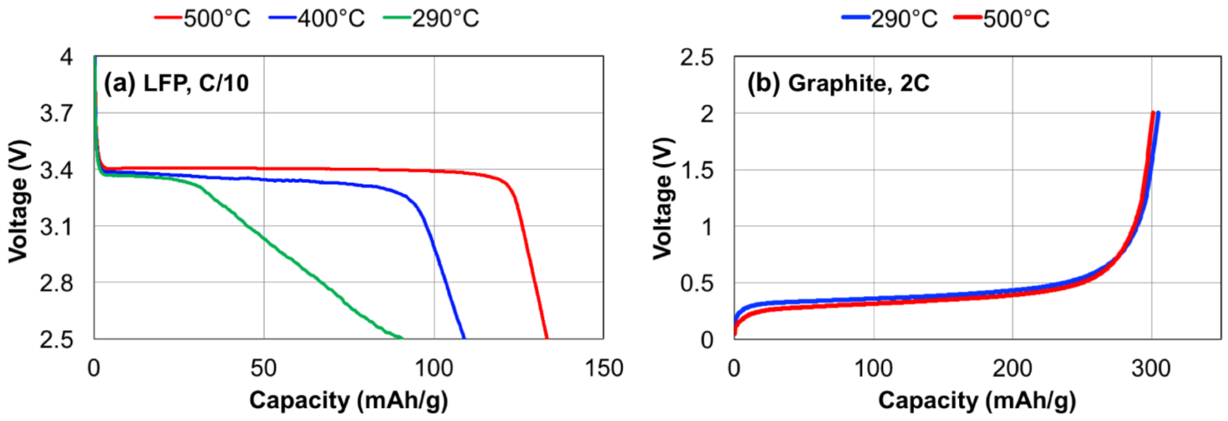

Supplementary Figure 3: Effect of heat treatment on silicate electrode lithium half-cell properties, for (A) lithium iron phosphate and (B) graphite.

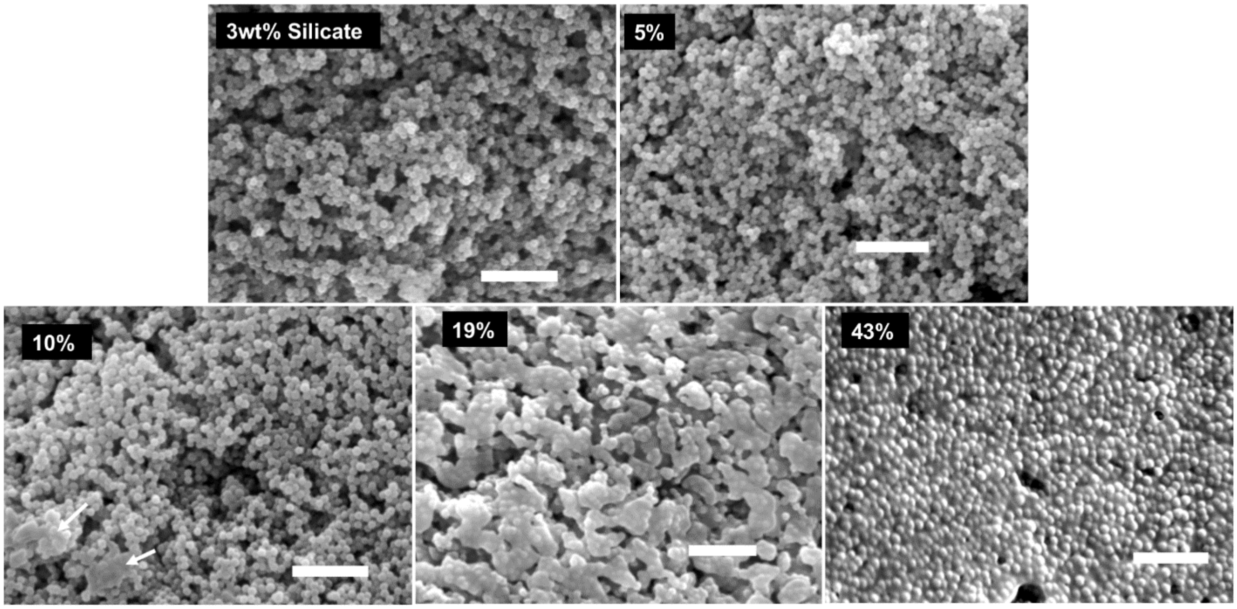

Supplementary Figure 4: Effect of separator paste composition on morphology at 3%, 5%, 10%, 19%, and 43% by weight silicate. The sample at 10wt% silicate exhibits the composition described in table S.E.1. This sample shows some aggregation of particles as highlighted by the arrows. All scale bars are 5 μm.

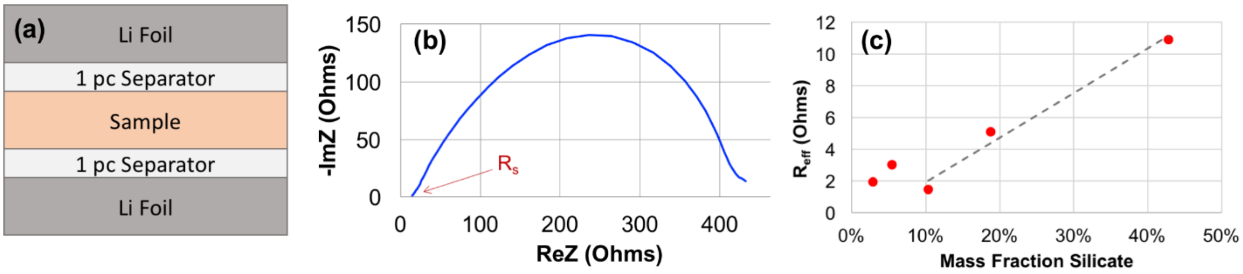

Supplementary Figure 5: The separator composition was measured using electrochemical impedance spectroscopy (EIS). A, Separator samples made as described in the text were loaded into CR2023 coin cells in the configuration shown. B, Control and test samples were tested using EIS, and the high-frequency intercept with the real axis was taken to be RS. C, R_{Eff} corresponding to Li+ ion transport through the sample was calculated and plotted as a function of separator composition.

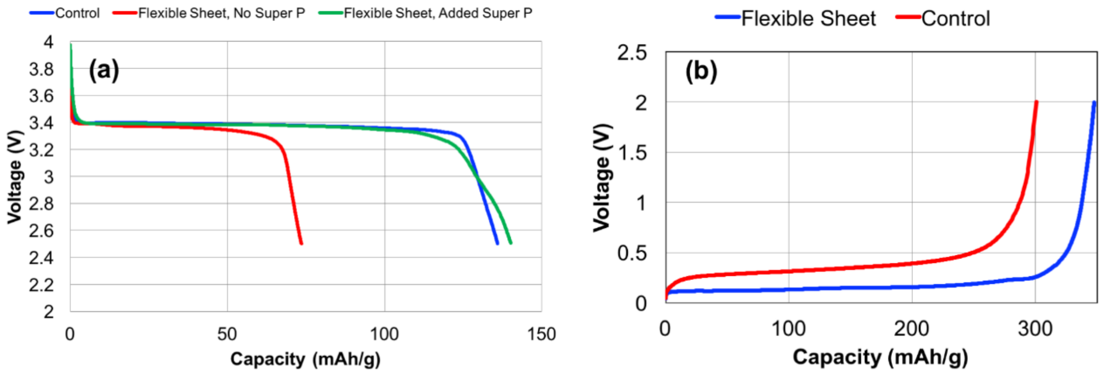

Supplementary Figure 6: Electrochemical performance of lithium half cells made using electrodes derived from flexible sheets, assembled into CR2023 coin cells as described in the Methods. A, Lithiation comparison at C/10 nominal rate of LFP half cells. B, delithiation comparison at 2C nominal capacity of graphite electrodes. Capacities are normalized by active LFP and Graphite content.

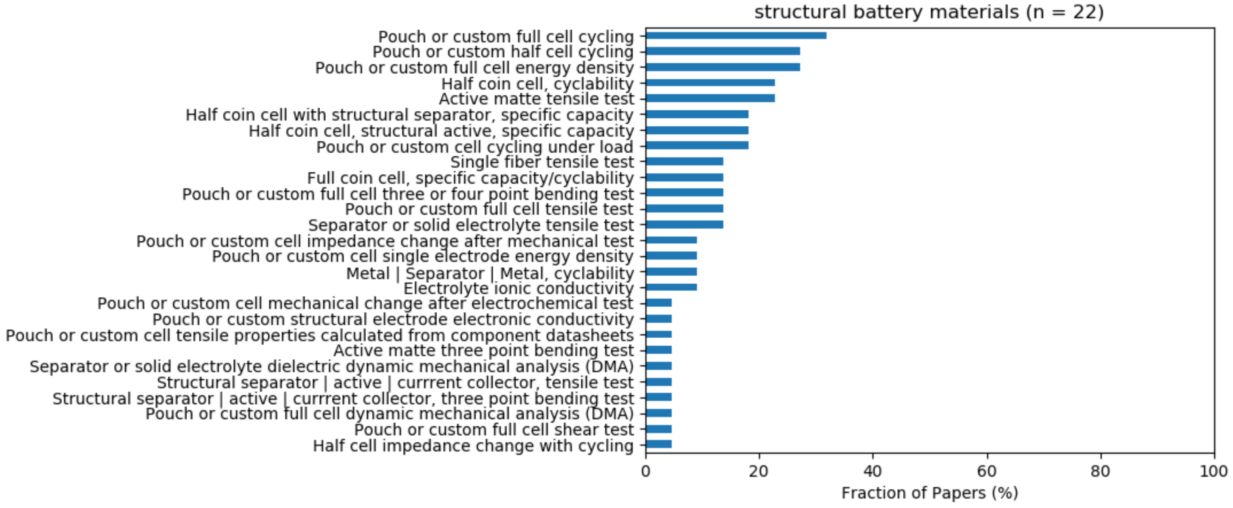

Supplementary Figure 7: Prevalence of reported metrics among structural battery materials

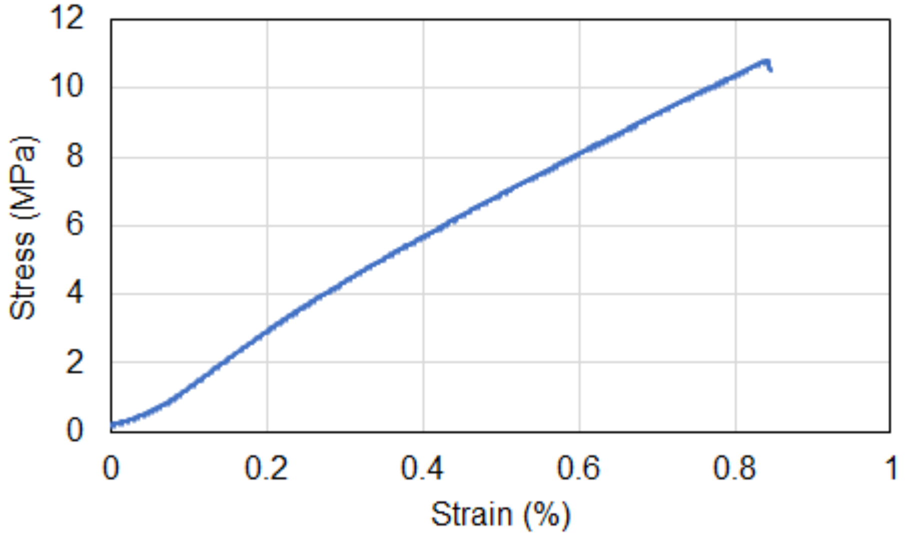

Supplementary Figure 8: Stress-strain curve of full SCB cell stack measured during tensile test.

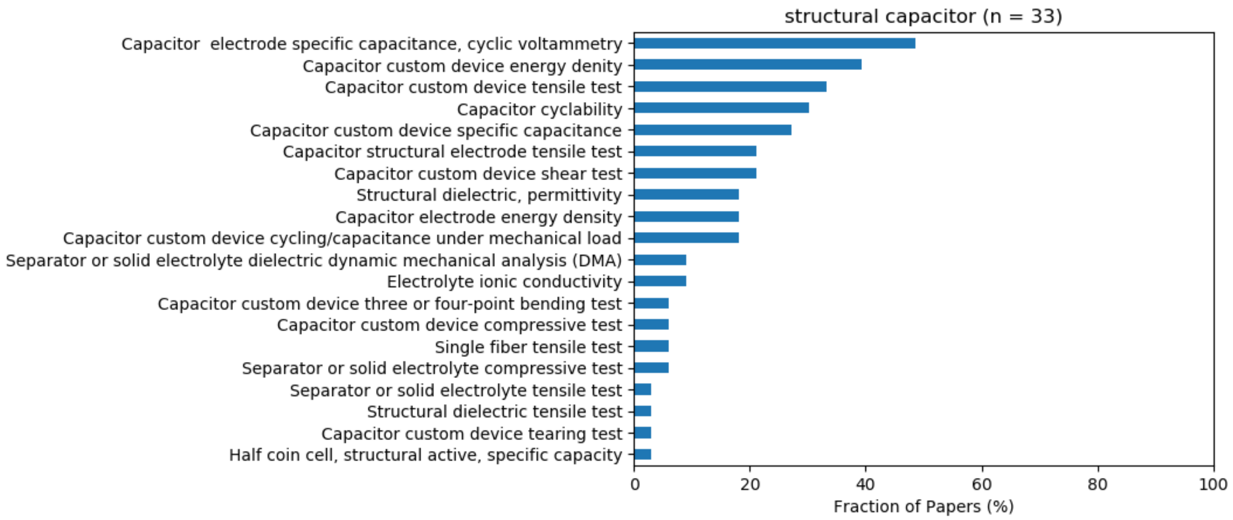

Supplementary Figure 9: Prevalence of reported metrics among structural capacitor materials

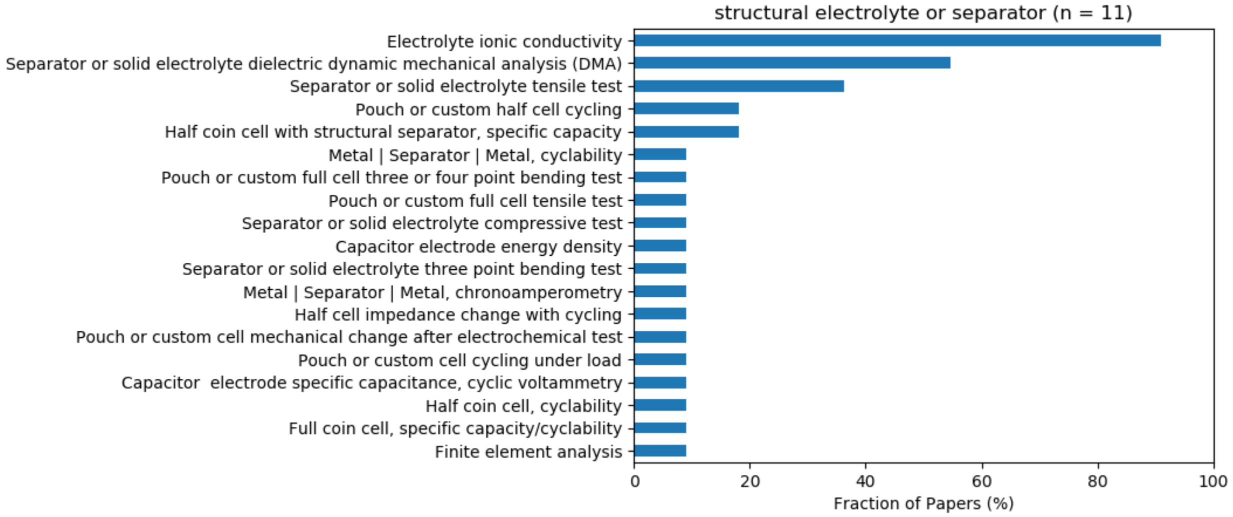

Supplementary Figure 10: Prevalence of reported metrics among structural electrolyte and separator materials

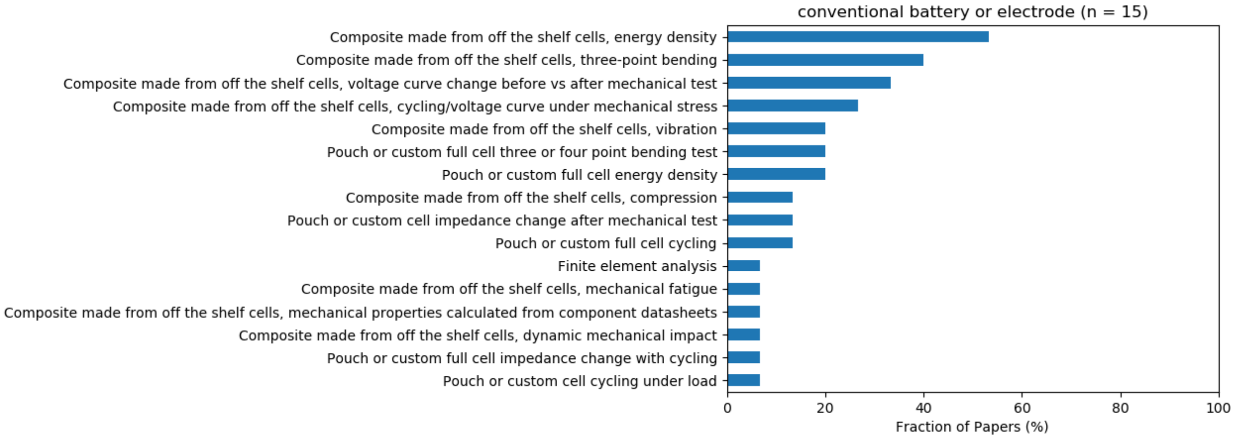

Supplementary Figure 11: Prevalence of reported metrics among structural energy storage devices incorporating batteries and electrodes with a conventional design.

Heat Treatment	Na/Si Atomic Ratio
None	1.8 +/- 0.6
500 ºC	0.54 +/- 0.16

Supplementary Table 1: Elemental ratios in untreated and heat treated samples measured using EDX.

Component	Wet Slurry	Dry Film	Sintered Separator
Silicate	2.7%	7.4%	10%
SiO2 Particles	22%	61%	85%
CMC	3.9%	11%	5%
Glycerol	7.8%	21%	0%
Deionized Water	82%	0%	0%

Supplementary Table 2: Base composition of separator paste

Publication	Energy Density (Wh/kg)	Energy Density (Wh/L)	Tensile Strength (MPa)	Tensile Modulus (GPa)	Active Chemistry
Asp, 2021[1]	23.6	-	300	25	LFP/Carbon Fiber
Thakur, 2020[2]	-	1.05	145	0.289	LFP/Carbon Fiber
Zhao, 2020 [3]	-	-	270	28	LFP/Carbon Fiber
Moyer, 2020[4]	58	-	33	-	
Moyer, 2020 [5]	37	-	213	0.0018	LFP/Carbon Fiber
Meng, 2018[6]	1.4	-	270	7	Ni Hydroxide / Iron Hydroxide
Ren, 2019 [7]	-	-	33	-	LFP/Li (half cell)
Ren, 2018 [8]	-	-	59	-	LFP/Na (half cell)
Javaid, 2018 [9]	-	0.4	-	-	LCO/Carbon Fiber
Nowak, 2017 [10]	-	-	628	37	LFP/Lignin-based Carbon Fiber
Yu, 2017 [11]	-	-	380	45	Carbon Fiber/Li (half cell)
Hagberg, 2016 [12]	-	-	3530-6370	230-588	Carbon Fiber/Li (half cell)
Westover, 2014	10	-	0.3	-	Silicon / PEO (capacitor)
Kim, 2012 [13]	-	-	-	360	Carbon Fiber/Li (half cell)
Liu, 2009 [14]	35	-	12	3.1	LCO/Graphite
Snyder, 2009 [15]	-	-	5520	965	Carbon Fiber/Li (half cell)

Supplementary Table 3: Summary of results from published multifunctional energy storage composite materials.

	Total Mass	Active	Liquid Electrolyte	Inactive	Porosity
LFP	43.6 mg/cm^2	53 m%	33 m%	14 m%	23 v%
Graphite	14.8 mg/cm^2	50 m%	34 m%	16 m%	27 v%
Separator	26.4 mg/cm^2	-	28 m%	72 m%	37 v%
**Total loading**	**84.8 mg/cm^2**				
Discharge energy	0.985 mWh				
Area Specific Discharge	7.97 mWh/cm^2				
**Energy density**	**93.9 Wh/kg**				

Supplementary Table 4: Total energy density calculations. Total loading of a layer includes active and inactive components of the solid electrode as well as electrolyte mass in the assembled cell. Layer component loadings are given as mass fraction (m%) of total layer mass.

Supplementary Methods

Nanoindentation

Nanoindentation was performed as described in the methods and Young's modulus values were compared to those of conventional PVDF binder as reported in the literature[16]. A heat treatment process was developed based on curing instructions for the use of commercial products using silicate as an adhesive binder[17] and described in the Methods section. In order to determine the effect of heat treatment on binder mechanical properties, samples treated at varying temperatures were measured. It was found that the Young's modulus of samples treated at 290ºC, the minimum temperature required to remove structural water, were softer and more variable than samples treated at higher temperatures. Samples treated at 400ºC and 500ºC exhibited a higher modulus. These results are shown in Supplementary Figure 1.

Transmission Electron Microscopy

Samples of silicate-LFP films made as described in the methods were examined. Electrodes with no heat treatment (Supplementary Figure 2A) were compared to electrodes heated to 500°C (Supplementary Figure 2B) so that the effect of heat treatment could be observed. During TEM investigations of the electrodes heated to 500°C, it appeared that crystalline silicate was not present. Evidence of ion exchange between LFP and waterglass was observed from elemental mapping in TEM (Supplementary Table 1). The amount of detectable sodium decreased with heat treatment by more than a factor of three. A likely explanation for this is that sodium present on the surface is exchanged with lithium in the iron phosphate. This is consistent with the fact that crystalline silicate was not observed, as ion exchange would be expected to entropically stabilize the amorphous silicate.

Effect of heat treatment on half-cell cycling

Rigid silicate electrodes were made as described in the methods, and heat treated in Argon atmosphere. A heat treatment to temperature T consisted first of a ramp up to 90 °C and a two hour hold, then a ramp up to temperature T and a two hour hold, then a ramp down to room temperature. All ramps were 30 min duration. Lithium half cells were made as described in the methods. LFP C/10 lithiation curves (Supplementary Figure 3A) and graphite 2C delithiation curves (Supplementary Figure 3B), based on nominal capacities as described in the methods, are shown. The LFP voltage profile is significantly affected by heat treatment temperature, while the graphite voltage profile is not.

Separator optimization

The SCB electrode composition described in the methods was used as a starting point for determining the separator paste composition, with 0.5 µm diameter SiO2 particles replacing active material particles. In order to determine the optimal separator paste composition, separators with varying silicate content were produced. The base composition for a 10 wt% silicate separator is described in Supplementary Table 2. The silicate loading was varied, maintaining a constant mass fraction of SiO2, CMC, and glycerol in the wet slurry. Slurries were coated onto a glass fiber tow and dried, resulting in a flexible film. These films were heated in argon (90ºC 2h, 500ºC 2h, all ramps 30 min) in order to sinter the separator and simulate the heat treatment process that an SCB undergoes during processing. Morphologies of samples with varied silicate composition are shown in Supplementary Figure 4. As the silicate composition is varied, samples up to 10 wt% silicate show very little aggregation of SiO2 particles. The morphology of these samples is largely a porous film of individual particles. At 10wt%, there begins to be some aggregation. The 19wt% sample is composed of 10-20µm aggregates of SiO2, while the 43wt% silicate sample is a uniform film of silica with particles embedded in it.

The resistance of these films was tested using electrochemical impedance spectroscopy (EIS). Cells were made using the configuration shown in Supplementary Figure 5A and measured as described in the methods. The solution resistance Rs was taken to be the high-frequency intercept of the Nyquist impedance plot with the real axis, as shown in Supplementary Figure 5B. Rs was measured for experimental samples as well as controls containing no sample. The resistance corresponding to transport through the separator was controlled for by calculating the effective resistance corresponding to transport through separator samples, taken to be: $R_{Eff} = R_{S,Sample} - R_{S,Control}$ This R_{Eff} is shown in Supplementary Figure 5C.

As shown in Supplementary Figure 5C, R_{Eff} is approximately constant for compositions under 10wt% silicate and increases approximately linearly from 10wt% to 43wt%. This is highly compatible with the separator morphology shown revealed by SEM micrographs. Sample morphology changes very little with the addition of silicate under 10wt%. The morphology in this case corresponds mainly to 0.5µm diameter SiO2 particles with very little apparent volume fraction devoted to silicate filler. At 10wt% we begin to observe aggregates of particles with a substantial volume fraction devoted to silicate. These electrochemical results suggest that at high volume fractions silicate, electrolyte is displaced and/or the tortuosity of the separator increases.

Electrochemical characterization of electrodes made from flexible sheets

Lithium half cells were made using both the rigid SCB electrode formulation and the flexible sheet formulation described in the methods. All samples were heat treated in Argon (90ºC 2h, 500ºC 2h, all ramps 30 min), then assembled into Lithium half-cells and tested as described in the methods. The LFP electrochemical results demonstrate that carbon nanofiber is not a sufficient electrical conductor in the LFP formulation to replace Super P and that the flexible sheet formulation results in positive electrodes with comparable discharge capacity to SCB electrodes produced using the rigid formulation

(Supplementary Figure 6A). The negative electrode electrochemical results, made without Super P, show that the carbon nanofiber is involved in lithium storage resulting in a ~17% increased capacity when both electrodes are normalized by the mass of the active graphite alone (Supplementary Figure 6B).

Active Stack Tensile Tests

The mechanical properties of the active stack were measured as described in the main text and methods, producing stress-strain curves as shown (Supplementary Figure 9). The active stack used for structural tests was identical to the composite material used for electrochemical tests, including structural elements such as the carbon nanofiber and glass fiber incorporated into the three layers of the active stack as described.

Survey of reported structural battery metrics

Categories of Structural Battery

In order to quantitatively assess the degree of comparability between metrics reported in the structural battery literature, a search for relevant papers was performed in Web Of Science. Firstly, the keywords: “multifunctional OR load-bearing” were searched for yielding 84,943 results. This was refined by topic: “battery OR capacitor”, yielding 1,592 results. Because many of the resulting papers dealt with non-mechanical multifunctionality, these results were further refined by topic: “mechanical”, yielding 291 papers. These results were manually reviewed, yielding 114 papers relevant to structural batteries and capacitors. This was supplemented through searches for other, more narrowly scoped keywords and following citations from reviews or other papers.

The resulting papers for which a full text could be obtained were sorted into the following categories:

- Structural battery materials with load-bearing electrodes (22 papers)
- Structural capacitors (32 papers)
- Structural electrolytes or separators (11 papers)
- Conventional batteries or electrodes used for structure (15)
- Remainder were excluded upon review based on topic, or because they did not introduce a new structural battery dataset based on laboratory tests

Reported metrics

Upon manual review, 55 commonly reported metrics and experimental datasets were found. Each paper was rated for each of these metrics. As the goal was to determine when a direct comparison between two materials was possible, metrics depend on both sample preparation and the measurement method used. Emphasis was given to metrics relevant to the structural energy storage performance of the materials presented.

Assessment of reported structural battery properties

As described above and in the main text, there is no consensus as to reporting metrics for structural battery properties. In particular, a wide range of standards were found for reporting energy densities. A summary of reported energy density and tensile strength metrics for structural battery materials is given in Supplementary table 3.

Cell stack component masses

Full cells were fabricated as described in the methods. The electrode and separator layers were punched from a flexible sheet produced as described in the methods. All layers were weighed as dry components before assembling. The post-sintering mass was determined by multiplying by the measured ratio of sintered to dry electrode sheets. The PEO loading was calculated by measuring the mass of the entire stack before and after the addition of PEO and assuming even distribution. Electrolyte mass was included in the electrode stack weight calculation (Supplementary Table 4). The discharge energy was calculated using BioLogic software.

Inactive materials included in each cell stack layer are as follows:

- Positive electrode: sodium silicate binder, Super P conductive carbon, carbon nanofiber, sodium carboxymethyl cellulose char, poly(ethylene oxide)
- Negative electrode: sodium silicate binder, carbon nanofiber, sodium carboxymethyl cellulose char, poly(ethylene oxide)
- Separator: sodium silicate binder, glass fiber, silica powder, chopped glass fiber, poly(ethylene oxide),

Supplementary References

1. Asp, L. E. *et al.* A Structural Battery and its Multifunctional Performance. *Adv. Energy Sustain. Res.* **2**, 2000093 (2021).

2. Thakur, A. & Dong, X. Printing with 3D continuous carbon fiber multifunctional composites via UV-assisted coextrusion deposition. *Manuf. Lett.* **24**, 1–5 (2020).

3. Zhao, Y. *et al.* Preparation and multifunctional performance of carbon fiber-reinforced plastic composites for laminated structural batteries. *Polym. Compos.* **41**, 3023–3033 (2020).

4. Moyer, K., Ait Boucherbil, N., Zohair, M., Eaves-Rathert, J. & L. Pint, C. Polymer reinforced carbon fiber interfaces for high energy density structural lithium-ion batteries. *Sustain. Energy Fuels* **4**, 2661–2668 (2020).

5. Moyer, K. *et al.* Carbon fiber reinforced structural lithium-ion battery composite: Multifunctional power integration for CubeSats. *Energy Storage Mater.* **24**, 676–681 (2020).

6. Meng, C. *et al.* Multifunctional Structural Ultrabattery Composite. *Nano Lett.* **18**, 7761–7768 (2018).

7. Ren, X. *et al.* Flexible Nanofiber-Reinforced Solid Polymer Lithium-Ion Battery. *Energy Technol.* **7**, 1900064 (2019).

8. Ren, X. *et al.* Iron Phosphate Coated Flexible Carbon Nanotube Fabric as a Multifunctional Cathode for Na-Ion Batteries. *Small* **14**, 1703425 (2018).

9. Javaid, A. & Ali, M. Z. Multifunctional structural lithium ion batteries for electrical energy storage applications. *Mater. Res. Express* **5**, 055701 (2018).

10. Nowak, A. P. *et al.* Lignin-based carbon fibers for renewable and multifunctional lithium-ion battery electrodes. *Holzforschung* **72**, 81–90 (2018).

11. Yu, Y. *et al.* Multifunctional structural lithium ion batteries based on carbon fiber reinforced plastic composites. *Compos. Sci. Technol.* **147**, 62–70 (2017).

12. Hagberg, J., Leijonmarck, S. & Lindbergh, G. High Precision Coulometry of Commercial PAN-Based Carbon Fibers as Electrodes in Structural Batteries. *J. Electrochem. Soc.* **163**, A1790 (2016).

13. Effects of carbon fiber electrode deformation in multifunctional structural lithium ion batteries - Hyon C Kim, Ann M Sastry, 2012.

https://journals.sagepub.com/doi/full/10.1177/1045389X12449914?casa_token=muBFM_7EIRIAAAAA%3AxNAYTz6q1dNbgGO-A7w9A3FTKdnBM7ToCrX7tavqRlME9N0iGTVQ2trSIhl9qCQmWs8Fi8sL5n8g-Q.

14. Liu, P., Sherman, E. & Jacobsen, A. Design and fabrication of multifunctional structural batteries. *J. Power Sources* **189**, 646–650 (2009).

15. Snyder, J. F., Wong, E. L. & Hubbard, C. W. Evaluation of Commercially Available Carbon Fibers, Fabrics, and Papers for Potential Use in Multifunctional Energy Storage Applications. *J. Electrochem. Soc.* **156**, A215 (2009).

16. Kovalenko, I. *et al.* A Major Constituent of Brown Algae for Use in High-Capacity Li-Ion Batteries. *Science* **334**, 75–79 (2011).

17. Ted Pella. PELCO High Temperature Carbon Paste, 50g, Technical Notes.
