# Supplementary material for: Structural ceramic batteries using an earth-abundant inorganic waterglass binder
Source: Nat Commun. 2021 Nov 11;12:6494. doi: 10.1038/s41467-021-26801-y (PMC8585950; doi:10.1038/s41467-021-26801-y)
Supplement: Supplementary file 2 — Description of Additional Supplementary Files [file 41467_2021_26801_MOESM2_ESM.pdf]

Description of Additional Supplementary Files

File Name: Supplementary Movie 1

Description: This video demonstrates handling of a freestanding SCB sheet before sintering.

The flexibility and robustness of these sheets enable the lay-up process described in the text.

File Name: Supplementary Data 1

Description: Data on metrics used to evaluate structural battery systems, compiled from literature review as described in the text and supplementary information.
